# ENDOCRINOLOGY IN THE TIME OF COVID-19: Diagnosis and management of gestational diabetes mellitus

**DOI:** 10.1530/EJE-20-0401

**Published:** 2020-05-26

**Authors:** Shakila Thangaratinam, Shamil D Cooray, Nithya Sukumar, Mohammed S B Huda, Roland Devlieger, Katrien Benhalima, Fionnuala McAuliffe, Ponnusamy Saravanan, Helena J Teede

**Affiliations:** 1 WHO Collaborating Centre for Women’s Health, Institute of Metabolism and Systems Research, University of Birmingham, Birmingham, UK; 2 Birmingham Women’s and Children’s, NHS Foundation Trust, Birmingham, UK; 3 Monash Centre for Health Research and Implementation, School of Public Health and Preventive Medicine, Monash University, Melbourne, Australia; 4 Diabetes Unit, Monash Health, Clayton, Australia; 5 Division of Health Sciences, Warwick Medical School, University of Warwick, Coventry, UK; 6 Department of Diabetes & Metabolism, St Bartholomew’s and Royal London Hospitals, Barts Health NHS Trust, London, UK; 7 Department of Obstetrics and Gynaecology, University Hospitals Leuven, Leuven, Belgium; 8 Unit Woman and Child, Department of Development and Regeneration KU Leuven, Leuven, Belgium; 9 Department of Endocrinology, University Hospital Gasthuisberg, KU Leuven, Leuven, Belgium; 10 UCD Perinatal Research Centre, School of Medicine, University College Dublin, National Maternity Hospital, Dublin, Ireland; 11 Academic Department of Diabetes and Metabolism, George Eliot Hospital, Nuneaton, UK

## Abstract

The COVID-19 pandemic has required rapid transformation and adaptation of healthcare services. Women with gestational diabetes mellitus (GDM) are one of the largest high-risk groups accessing antenatal care. In reformulating the care offered to those with GDM, there is a need to balance the sometimes competing requirement of lowering the risk of direct viral transmission against the potential adverse impact of service changes. We suggest pragmatic options for screening of GDM in a pandemic setting based on blood tests, and risk calculators applied to underlying risk factors. Alternative models for antenatal care provision for women with GDM, including targeting high-risk groups, early lifestyle interventions and remote monitoring are provided. Testing options and their timing for postpartum screening in women who had GDM are also considered. Our suggestions are only applicable in a pandemic scenario, and usual guidelines and care pathways should be re-implemented as soon as possible and appropriate.

## Introduction

The SARS-CoV-2 viral pandemic has had a profound impact on health service provision internationally, including on antenatal care (1). Impacts vary according to national health resources, models of antenatal care, severity of the pandemic and regional containment policies and by the ability of the healthcare system to rapidly adapt and transform. The direct health impact of the pandemic on routine antenatal care, particularly for high-risk pregnancies, is critical to consider. Women with gestational diabetes (GDM), a condition defined by glucose intolerance diagnosed for the first time in pregnancy (2), are one of the largest groups of high-risk women accessing antenatal care in the hospitals on a frequent basis (3, 4, 5), due to increased risk of adverse pregnancy outcomes (6). While efforts to limit viral transmission through physical distancing and minimizing the strain on redeployed healthcare resources are important, it is crucial to recognize and plan for disruption to routine antenatal screening, detection, monitoring, prevention and management and subsequent adverse health outcomes for women with GDM (1, 7). Importantly, healthcare providers must be aware of the inequitable impact of crises and health service limitations and consider this in personalised care (7).

Outside the pandemic scenario, screening for GDM with an oral glucose tolerance test (OGTT) is offered either to all women (universal screening) or based on risk factors (selective screening), and the criteria used to diagnose GDM vary (8, 9). The OGTT usually involves long waits at hospital and involves a dedicated phlebotomy service. The test has a high sensitivity (low false negative rate) for GDM diagnosis (10, 11). Due to concerns that universal OGTT screening using the International Association of Diabetes and Pregnancy Study Groups (IADPSG) criteria overdiagnoses GDM without clear clinical benefit (12), many European guidelines recommend risk factor-based screening (13). Once GDM is diagnosed, usual antenatal care involves frequent face-to-face consultations with diabetes and/or obstetric teams, lifestyle and potential pharmacological interventions requiring multiple visits, and 4-weekly scans to monitor fetal growth. Additional postpartum visits for tests (OGTT, fasting plasma glucose (FPG) or HbA1C) are required to detect conversion to type 2 diabetes (T2D) in women with GDM (9, 14).

Usual evidence-based approaches in developing new models of care that ideally require stakeholder engagement, co-design and evaluation are all seriously curtailed during the pandemic. Therefore, at this time pragmatic changes are required to all stages of GDM care including screening and diagnosis, management and follow-up. These must balance the need to prevent GDM-related complications against limiting the risk of SARS-CoV-2 virus transmission to mothers in overstretched health systems. We now explore this balance and suggest risk mitigation strategies allowing identification of women with overt diabetes early in pregnancy, and risk of GDM through alternative screening approaches and modified pathways for monitoring, management and follow-up. These represent general suggested approaches. They will require tailored implementation strategies that may vary by country, by baseline local GDM management practices and by regional pandemic severity. Our recommendations are only in the context of the pandemic, and we emphasize that diagnosis and care should realign to current guidelines when this is safe and feasible.

## Screening for gestational diabetes mellitus in an evolving pandemic

**Figure 1:**
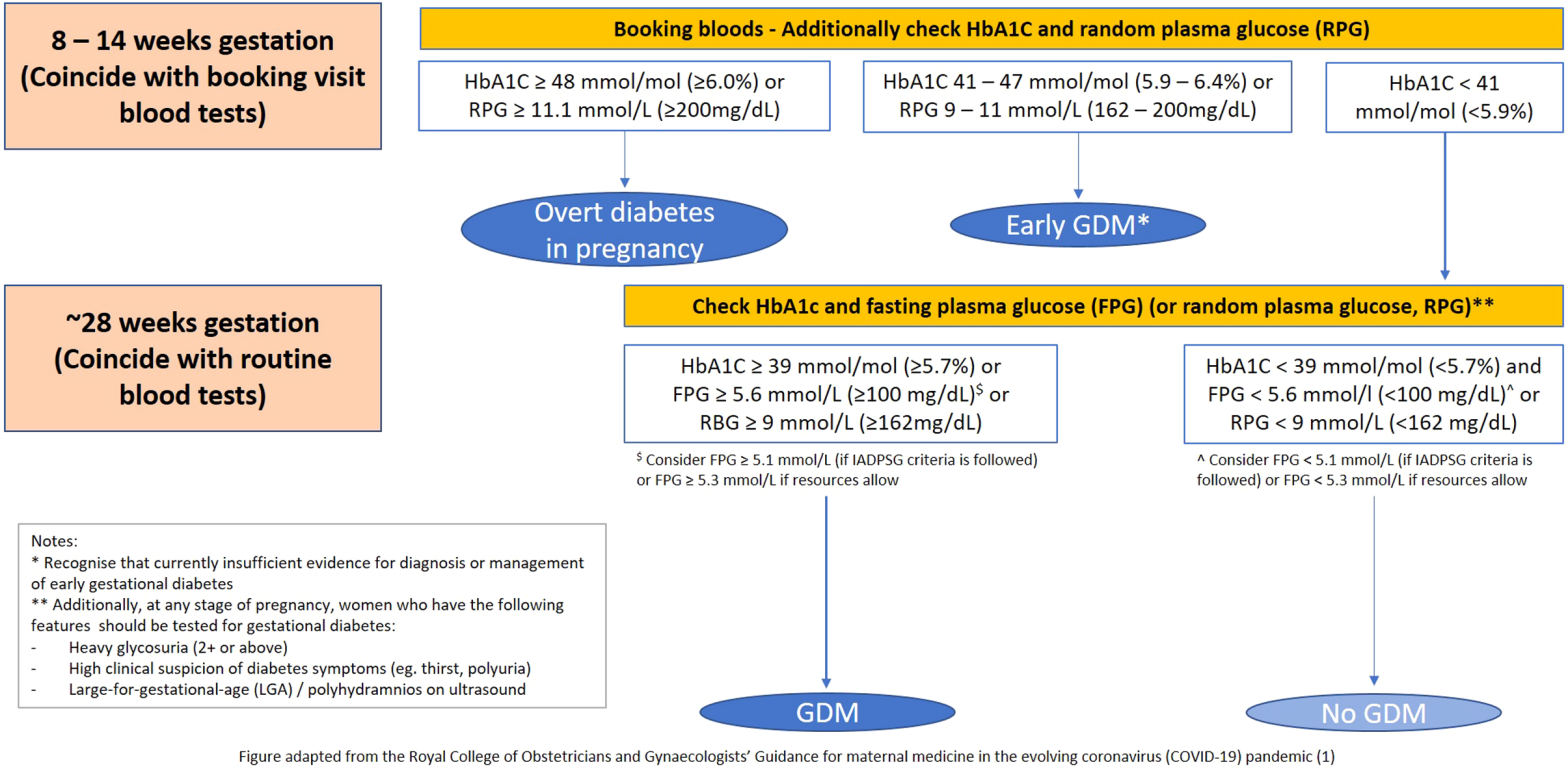
Screening for gestational diabetes mellitus in women with risk factors during the evolving COVID-19 pandemic. Figure adapted from the Royal College of Obstetricians and Gynaecologists’ Guidance for maternal medicine in the evolving coronavirus (COVID-19) pandemic (1).

In an evolving pandemic with a highly infectious virus, screening OGTTs involve high exposure risks and health service burden. The routine use of OGTTs for GDM screening needs to be carefully considered in the context of local pandemic impact including community transmission rates. Where it is no longer safe or feasible current evidence does not support a single alternative test. We thus propose a strategy that utilizes alternative simpler tests and mitigation ‘safety-nets’ balancing GDM detection with minimizing of health service burden and viral exposure of women (Fig. 1):

- Undertake additional tests at booking (HbA1C and random plasma glucose (RPG)), primarily to detect overt diabetes, and identify those at highest risk for GDM;
- Avoid OGTT at 24–28 weeks and instead offer HbA1C along with fasting plasma glucose (FPG) or RPG;
- Identify and monitor women with past GDM;
- Apply a personalized risk calculator for universal screening of GDM;
- Monitor for complications of any GDM missed in routine care; and
- Real-time evaluation of the impact of pandemic screening strategy implementation.

We recommend that centers follow their current guidelines on whom to screen (selective or universal). For centers who currently use universal screening and feel this to be unsafe during the pandemic, we recommend using selective screening in conjunction with our proposed testing strategy. We have taken into account the following in providing suggestions for GDM screening in a pandemic: feasibility in an overstretched healthcare environment; minimizing travel, number of visits and duration of exposure for screening; characteristics of the screening tests. Highly sensitive tests (low false negative rate) with low specificity will increase the numbers of false positives and burden the system, while highly specific tests (low false positive rate) with low sensitivity may miss GDM, risking adverse outcomes for mother and fetus. Given the risk that some GDM may not be detected with the proposed changes in screening strategy, all women should be encouraged to follow a healthy lifestyle including diet throughout pregnancy (15, 16).

### 1. Early screening for GDM

Early screening for GDM is primarily designed to identify women who had undetected diabetes before pregnancy, given the risks of undiagnosed (largely type 2) diabetes in pregnancy, but there is no universal or risk factor-based screening in the first trimester in many countries in normal times. In the UK, the NICE guidelines recommend OGTT in first or early second trimester for women with previous GDM to aid early detection (9). Our proposal for early screening in a pandemic aims to identify women who would otherwise be diagnosed with abnormal glucose concentration only at 24–28 weeks of pregnancy.

At booking, HbA1c and RPG can be performed in addition to usual booking bloods, to detect the highest risk groups. We suggest the following thresholds and actions:

- **HbA1c ≥48 mmol/mol (≥6.5%) *or* RPG ≥11.1 mmol/L (≥200 mg/dL): treat as pre-existing diabetes.** HbA1c ≥48 mmol/mol (≥6.5%) in non-pregnant adults is universally accepted for diagnosing overt diabetes (17, 18), and HbA1c tends to fall slightly in pregnancy. In interpreting results, consideration of individual risk for T2D is advisable. Care should be taken where HbA1c is unreliable, such as in beta thalassemia and severe anemia (19, 20). As the recommended antenatal routine booking blood tests are not taken in the fasting state, we suggest pragmatic use of RPG. A threshold of ≥11.1 mmol/L (≥200 mg/dL) is aligned with diagnostic criteria in the non-pregnant population (17, 21).
- **HbA1c 41–47 mmol/mol (5.9–6.4%) *or* RPG 9–11 mmol/L (162–200 mg/dL): consider managing using the GDM pathway:** An HbA1c between 41–47 mmol/mol (5.9–6.4%) confers risk for future T2D in non-pregnant women (17, 22). Our suggestions are based on the need to avoid unnecessary strain to the healthcare system through use of unduly low diagnostic cut-offs. We suggest that early GDM could also be diagnosed by an RPG of 9–11 mmol/L (162–200 mg/dL) for pragmatic reasons. RPG is considered a better predictor of GDM at 24–28 weeks than body mass index alone (23).

### 2. Screening blood tests for GDM at 24–28 weeks

Screening for GDM with OGTT is widely accepted at 24–28 weeks’ gestation, and the majority of evidence on diagnosis and treatment benefits is based on this. OGTT strategies and diagnostic thresholds vary internationally, however. During the pandemic, HbA1c, FPG, or RPG if fasting values are not available, offer a pragmatic alternative. In taking this approach, we suggest the following thresholds:

**HbA1c ≥39 mmol/mol (≥5.7%) or FPG ≥5.6 mmol/L (≥100 mg/dL) or RBG ≥9 mmol/L (≥162 mg/dL): treat as GDM.** Using FPG alone will only pick up about half of all women with GDM, based on NICE or IADPSG criteria (1). Combining FPG with HbA1C may improve the detection rate. We consider this reasonable and pragmatic, as women who are negative for GDM on OGTT (IADPSG criteria) and have high HbA1c at delivery (≥39 mmol/mol, ≥5.7%) are at higher risk of maternal and offspring adverse outcomes (24). The proposed combination of FPG and HbA1c criteria are expected to identify a similar number of women accessing health services with GDM, to current practice of NICE diagnosed GDM with OGTT. Rates of short-term adverse pregnancy outcomes (LGA, stillbirth, preterm delivery and cesarean section rates) are broadly similar (1). Maintaining existing FPG thresholds may be preferable, and services may consider lower thresholds consistent with the IADPSG diagnostic criteria (FPG ≥5.1), if resources allow.

### 3. Diagnosis and monitoring in women with past GDM

Women with a history of GDM have a high risk of subsequent GDM and of T2D. The risk of GDM in a subsequent pregnancy is approximately 50%, or up to 80% in some series (25, 26, 27). We therefore suggest that booking blood tests should be completed as outlined above, to detect pre-existing overt diabetes or early GDM where HbA1c, FPG or RPG thresholds are exceeded. If resources allow, healthcare services may consider regular glucose monitoring of women with a history of GDM, without the need for testing at 24–28 weeks, combined with early lifestyle interventions.

**Figure 2:**
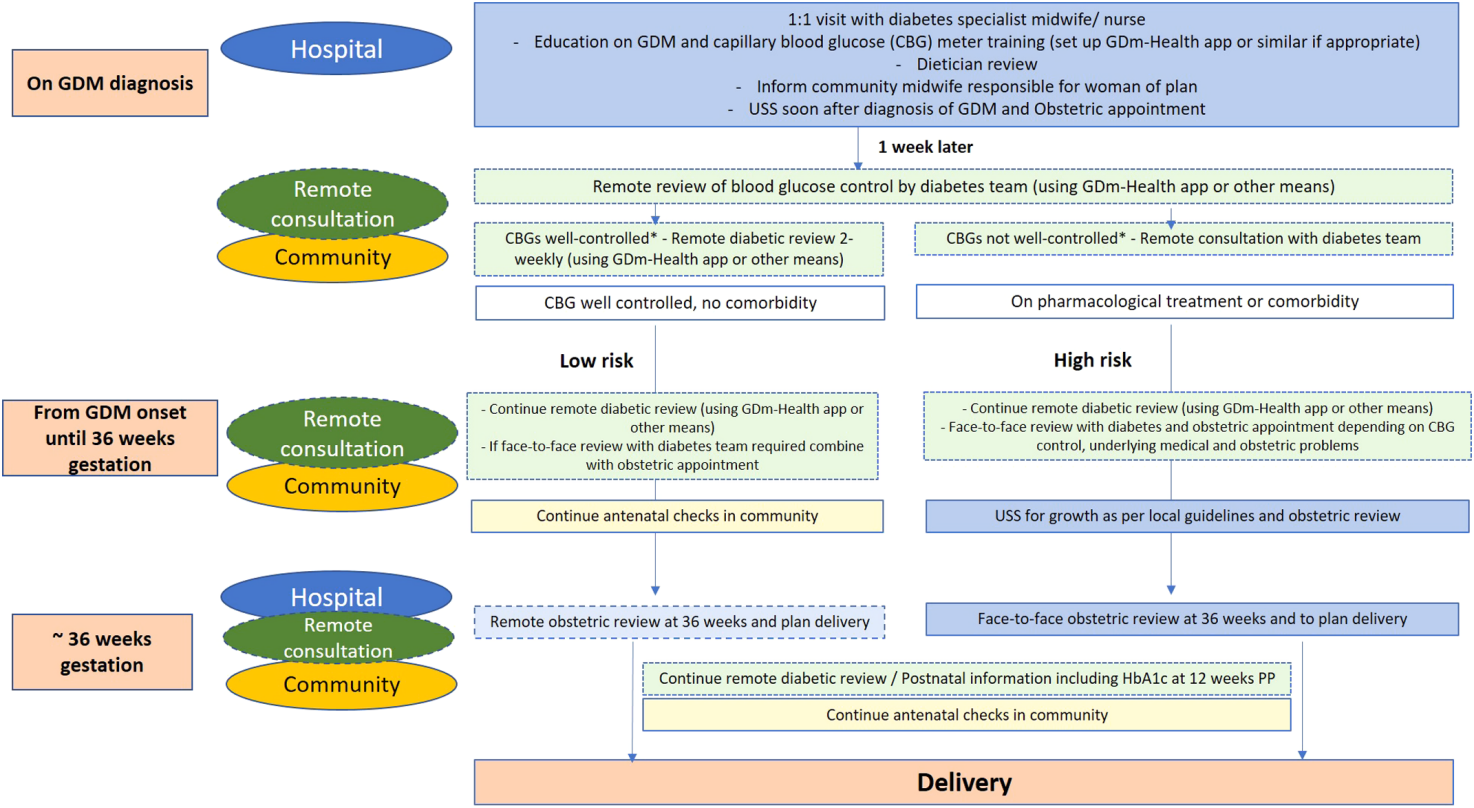
Management of women with GDM – the suggested patient pathway after a diagnosis of GDM during the COVID-19 pandemic. Provision of antenatal care in the community or hospital will depend on local healthcare systems and set-up of community (or equivalent healthcare) teams. *Well-controlled as per local guidelines. GDM, gestational diabetes mellitus; CBG, capillary blood glucose; USS, ultrasound scan; PP, postpartum.

### 4. Application of personalized risk calculators for universal screening of GDM

Risk calculators enable assessment of personal risk of developing GDM, and many have been externally validated (28). Of these, the Monash risk calculator has been robustly developed, internally and externally validated, integrated into a simple accessible online tool, undergone implementation research, has been implemented into routine care and is being integrated into national guidelines in the Netherlands (28, 29, 30, M P H Koster, personal communication). It includes clinical characteristics routinely collected at booking (maternal age, body mass index, ethnicity, previous GDM, family history of diabetes). This risk calculator has good discrimination (C-statistic 0.77; 95% confidence interval 0.73−0.81) and is well calibrated (slope 1.1) for predicting GDM (28). Due to the likely prolonged nature of the pandemic, although this may be a new practice for many services across the world, it is simple to implement, is evidence based and is worthy of consideration here and moving forward.

### 5. Ongoing monitoring for complications of any GDM missed in routine care

We recommend that women identified as having any of the following features at any time during pregnancy are screened for GDM, re-screened if they have previously had a normal GDM screening test, or commence routine glucose monitoring as per GDM protocols: heavy glycosuria (≥2+ glucose); symptoms of diabetes (e.g. thirst, polydipsia/polyuria, nocturia); large-for-gestational-age fetus or polyhydramnios on ultrasound. It has been shown that accelerated fetal growth occurs well before diagnosis of GDM (31) and increased abdominal wall thickness as a surrogate for fetal adiposity is seen at least 4–5 weeks prior to the biochemical diagnosis of GDM (32). However, this needs further evaluation as a screening test for GDM and services could conduct real-time audit for complications that may be due to missed GDM.

### 6. Real-time evaluation of the impact of pandemic GDM strategy implementation

With sophisticated real-time data sharing and analysis increasingly available, evaluation of the impact of these pandemic strategies is both feasible and vital, to iteratively monitor and improve outcomes.

## Models of glycemic and antenatal care in an evolving pandemic

Antenatal care for pregnant women with GDM should, wherever possible, minimize the need for hospital-based care, support public health measures concerning physical distancing and self-isolation and reduce health service burden. Women at high risk of GDM, such as those with previous GDM, or those identified on a risk prediction tool, could be offered early dietary and lifestyle advice (via remote delivery including telehealth consultations). While the individual RCTs show mixed results, overall these interventions are effective in limiting excess gestational weight gain (GWG) and our IPD shows (59 studies; *n* = 16885) that it can prevent GDM and its complications (33, 34). Once GDM has been diagnosed, education to facilitate healthy diet and physical activity is important. Healthy GWG, control of blood glucose concentration, and monitoring, prevention and management of GDM-related complications should remain the cornerstone of antenatal GDM management (9, 35). Centres with traditional face-to-face GDM education sessions are encouraged to transition to remote delivery, using mobile health tools, interactive webinars and online resources. However, after the pandemic, patient and public engagement with health professionals will be needed to co-design the optimal approach to lifestyle intervention in high-risk pregnancies and GDM. Best-quality evidence, including on cost effectiveness and implementation will be needed to guide delivery at scale.

After initial education and commencement of lifestyle measures, antenatal care in GDM should be stratified by risk of adverse pregnancy outcomes (36). Pragmatic stratification considers adequacy of blood glucose control, need for pharmacologic treatment and the presence of additional risk factors for adverse pregnancy outcomes such as obesity. We consider women with GDM on diet, with well-controlled blood glucose levels and no other risk factors to be at low risk of adverse pregnancy outcomes. We propose that these women are managed in routine antenatal care pathways, potentially in the community, if aligned with regional models of care (Fig. 2). Women with GDM requiring treatment with metformin and/or insulin (depending on local protocols), or those with other identified risk factors, are considered to be at higher risk of adverse pregnancy outcomes than those on diet alone. We propose that such women are routinely reviewed by the specialist diabetes team. Where usual practice involves frequent face-to-face reviews, a transition to remote review with a focus on assessing glycemia and therapy titration should be considered. Digital blood glucose monitoring systems with demonstrated safety and non-inferior efficacy such as GDm-health (37) or other similar local solutions may be a useful adjunct and can maximize women’s satisfaction with care. Where glycemic targets have been met, and in the absence of other risk factors for adverse pregnancy outcomes, remote obstetric review at 36 weeks allows planning for delivery. Where glycemic targets are not met, or where there are other risk factors for adverse pregnancy outcomes, face-to-face obstetric review may be considered. Similarly, inability to achieve glycemic targets may be an indication for serial growth scans with timings adapted to existing local practices.

## Postpartum screening for persistent diabetes in an evolving pandemic

Postpartum screening for persistent diabetes (T2D, maturity-onset diabetes of the young (MODY) or, less commonly, type 1 diabetes (T1D)) can occur immediately postpartum with either FPG or 24-h capillary blood glucose testing in those at high risk (increased BMI, high insulin dose in pregnancy and high-risk ethnicity). Postpartum OGTTs should ideally be delayed until public health measures for control of the pandemic have been eased. More timely postpartum screening for persistent diabetes during the pandemic could be considered where there are significantly increased risk factors for T2D, concerns for T1D or MODY, or where a woman is planning an early subsequent pregnancy, in which case pre-conceptional diagnosis would modify care. In this setting an HbA1c at 3–6 months postpartum could be completed in the community.

## Conclusion

We suggest pragmatic options for GDM screening, diagnosis and management during the pandemic, with integration into background routine antenatal care. We highlight that risk-stratified approaches can enable personalized care for individual women, and note that inequity and health disparities are amplified during a crisis, requiring recognition and consideration in providing care. Adapted GDM screening and diagnostic strategies and antenatal management pathways during the pandemic need to be considered in the context of local practice and iteratively evaluated and improved, both at the individual and at the population level, through analysis of real-time outcome data for women and their offspring. Evaluation should focus on changes to care prompted by the pandemic to avoid harm, capture high-value elements and inform post-pandemic care.

## Disclaimer

Due to the emerging nature of the COVID-19 crisis this document is not based on extensive systematic review or meta-analysis, but on rapid expert consensus. The document should be considered as guidance only, and it is not intended to determine an absolute standard of medical care. Healthcare staff need to consider individual circumstances when devising the management plan for a specific patient.

## Declaration of interest

H J T was the lead researcher of the study that developed the Monash risk calculator. S T, P S and M S H were involved in the development of the RCOG guidelines on antenatal care of pregnant women with diabetes in the context of the evolving pandemic. The other authors have nothing to disclose.

## Funding

K B and R D are the recipient of a ‘Fundamenteel Klinisch Navorserschap FWO Vlaanderen’. S D C is supported by a National Health and Medical Research Council http://dx.doi.org/10.13039/501100000265 (NHMRC) Postgraduate Scholarship, a Diabetes Australia http://dx.doi.org/10.13039/501100000971 Research Program NHMRC Top-up Scholarship, the Australian Academy of Science http://dx.doi.org/10.13039/501100000969’s Douglas and Lola Douglas Scholarship and an Australian Government http://dx.doi.org/10.13039/100015539 Department of Education and Training http://dx.doi.org/10.13039/501100007912 Endeavour Research Leadership Award. H J T is supported by an NHMRC Fellowship funded by the Medical Research Future Fund. The funding bodies had no role in the study design, the collection, analysis and interpretation of the data, the writing of the report, nor the decision to submit the paper for publication.

## Author contribution statement

Conceptualization: S T, P S, M S B H; Writing – original draft: S D C, S T; Writing – review and editing: All authors.
